# **Global Health in Undergraduate Education: Knowledge, Attitude, and Practice of Sudanese Medical Students towards Global Health Education: a cross-sectional study**

**DOI:** 10.1186/s12909-023-04168-6

**Published:** 2023-04-11

**Authors:** Sawazen Malik, Asi Alkoronky, Mugahid Elmahi, Safaa Alsafi, Fares Shehada, Rawasi Rahma, Daffalla Alam Elhuda

**Affiliations:** 1 Faculty of Medicine, University of Khartoum, Khartoum, Sudan; 2 Faculty of Medicine, Gezira University, Wad Madani, Sudan; 3 Faculty of Medicine, Alzaiem Alazhari University, Khartoum North, Sudan; 4 Faculty of Medicine, Omdurman Islamic University, Omdurman, Sudan; 5 Faculty of Medicine, Kordofan University, Al-Ubayyid, Sudan; 6 Academy of Health Sciences, Khartoum, Sudan

**Keywords:** Knowledge, Attitude, Practice, Global health, Education, Medical students

## Abstract

**Introduction:**

Globalization and other relevant phenomena such as healthcare workforce, ageing of the population, brain drain etc. all necessitate medical curricula to transcend national medicine and encompass a more global approach. This is especially true in the context of developing countries which currently act as passive recipients of ongoing global decisions, health inequities or pandemics. The aim of this research was to study the Knowledge, Attitude, and Practice of Sudanese medical students towards global health education and the impact of extra-curricular activities on their knowledge and attitudes.

**Methods:**

This was a descriptive cross-sectional institutional based study. The study was conducted in five Sudanese Universities and participants were selected using systematic random sampling. An online-based, self-administered questionnaire was used, samples were collected between November 2019 until April 2020 and data was analyzed using SPSS version 25.

**Results:**

1176 medical students were involved. The study revealed a poor level of knowledge among 72.4%, and only 2.3% of respondents revealed a good level of knowledge. Mean knowledge scores between Universities slightly vary and have shown positive correlation according to the grade of the medical student. Regarding attitude, the results demonstrated the high level of interest of medical students in global health, their agreement in including global health in their official medical education curriculum (64.8%) and their consideration of including global health as part of their future career (46.8%).

**Conclusion:**

The study concluded that there is a knowledge gap among Sudanese medical students regarding global health education, although students showed good attitudes and willingness to include global health in their official curriculum.

**Recommendations:**

Global health education should be implemented in the official curriculums of Sudanese Universities, and Universities should do global partnerships and increase the learning and teaching opportunities in this interesting field.

## Introduction

Global health is “an area for study, research, and practice that prioritizes improving health and achieving equity in health for all people worldwide. It emphasizes transnational health issues, determinants, and solutions that involve many disciplines within and beyond the health sciences and is a synthesis of population-based prevention with individual-level clinical care. Although global health prioritizes prevention, it also embraces curative, rehabilitative, and other aspects of clinical medicine and the study of basic sciences [1].”

Globalization and other relevant phenomena such as the healthcare workforce, aging of the population, and brain drain necessitate medical curricula to transcend national medicine and encompass a more global approach. If doctors are to be experts in health, a broad understanding of health worldwide and of diverse populations is essential and has political potency because improving global health could support national, regional, and global security interests by fostering political stability [2, 3]. Medical education in Sudan began in 1924. A 2005 study estimated that 60% of Sudanese physicians’ practice outside of the country since the 1960s [4] The causes of medical emigration are mostly financial, coupled with a lack of advanced training and career development opportunities. [5] in addition to active recruitment of physicians from Sudan to work abroad, generating a thriving diaspora of Sudanese practitioners. In his commentary, Eichbaum [6] argued that improving global health competencies necessitates the inclusion of health professionals in resource-limited settings. This calls for more input from developing countries, as current global health education literature is primarily dominated by findings and opinions from developed countries.

Global health education has various effects on medical students. For example, students who attended a voluntary HIV session were more willing to treat HIV patients locally after the intervention. [7] Similar cohort studies had the same results. Global health education groups participating in an elective in India were more likely than controls to report providing care to underserved populations. [8] The group was also more likely to report ongoing global health, public health, or public policy involvement. They deduced that individuals interested in global health experiences contribute to U.S primary care workforce and display cost-conscious attitudes that are essential to the practice of medicine in the U.S.

First-hand exposure is the most comprehensive educational experience for medical students [8–11]. Sudanese medical schools have included an overview of some global health topics, such as health determinants and universal health coverage, throughout the community medicine curriculum. Many schools have a 1-2-week rural residency program that exposes undergraduate students to rural communities and local health issues such as the double burden of communicable and non-communicable diseases. The concept of community-oriented medical education was first introduced in 1975 at Gezira University which emphasizes community medicine, and rural exposure is emphasized through additional rural visits with more diverse programs, as well as a well-integrated primary health exposure. Furthermore, extracurricular activities enable students and medical graduates to provide healthcare to vulnerable communities; for example, 7-day biannual medical missions are a well-established extracurricular program at Khartoum University. These experiences are associated with an increased interest in international and global health careers. However, this approach could have a harmful effect if it is not controlled, as a fruitful global health experience could diverge into ‘medical tourism’. [12] Other research found no association between global health education (GHE) and interest in global health careers. [10] Prior interest in global health is a much stronger factor than the duration of the elective. Demographic dimensions are also prominent influences such as increasing age, low household income, and female sex. [13] In addition, the delivery of global health as part of the humanity block was an opportunity to expose students to the social determinants of health through interdisciplinary learning [14]. However, all these studies derive from findings in high-income countries or a high-income country-based facility.

An International Federation of Medical Students Association (IFMSA) study [15] conducted among medical students found that 91% of respondents thought global health and women and child health should be a component of medical school curricula. They also highlighted that few medical students who received interdisciplinary teaching also received the lowest exposure, especially in low-middle-income countries (LMICs).

A comparison of global health elective experience among high-income countries (HICs) and LMICs showed that both groups had similar reasons for participation, including opportunities to experience different healthcare systems, resource difference settings, and the culture of medicine in different contexts. LMICs were more likely to report didactic experiences, electronic medical records exposure, and library skills, whereas U.S students were more likely to report outpatient clinical outcomes as important. Finally, students from LMICs were less likely to note differences in quality and access to healthcare based on social determinants of health. Overall, the study concluded that LMIC students were less likely to note what is considered fundamental tenets of global health education [16].

Similarly, Singh & Purohit [17] and Braimoh & Odai [18] showed that many students in India and Nigeria lacked knowledge of global health. For example, in Omigoberi and Emoka’s study, few students knew about FDI and the World Health Organization’s (WHO) global oral health policy and intervention. In Braimoh’s study, 95% did not know the meaning of Basic Package of Oral Care (BPOC), and less than a quarter knew that BPOC was created by the WHO. However, both studies showed that all the students wanted to volunteer in international settings, which is very different from findings in developed countries. Though, it is questionable whether this is an exacerbation of brain drain or a desire to help their communities in the future.

### Problem Statement

There is a paucity of research in global health and medical education from developing countries and Sudan, and findings from developed countries do not apply to our settings. For example, attitudes towards global health differ between Nigerian medical students and U.S. students. Global health education is necessary for the undergraduate experience. It is no longer adequate for students to learn national medicine and be divorced from the global status quo. Without this type of education, it is seldom expected that the curriculum will transform students into reliable health experts and health leaders. Currently, global health as a discipline is absent from Sudanese universities’ medical school curriculum; however, variant topics are disseminated throughout the teaching curriculum of community medicine, thus students must acquire their global health information and awareness from sources external to the official curriculum.

### **Justification**

The need for global health has not been greater. Africa is burdened by communicable and non-communicable diseases, health inequities, and phenomena such as brain drain. The rural population, which constitutes a large part of the Sudanese population (65.99% in 2017), is greatly underserved. If medical graduates lack awareness or the knowledge and inspiration to combat this, the problem is exacerbated. Global health education is a significant factor involved in this; hence, the lack of knowledge and poor attitude of students towards this discipline have implications not only for medical students educationally but also for the country and continent. These factors prompted the genesis of this research, which is the first to be conducted in Sudan and aims to study the knowledge, attitude, and practice of Sudanese medical students towards global health education and the impact of extra-curricular activities on their knowledge and attitudes.

## Research Methodology

### 1- Study design

This was a descriptive cross-sectional institutional-based study conducted in five governmental Sudanese universities: Khartoum University, Gezira University, Omdurman Islamic University, Alzaiem Alazhari University, and Kordofan University. Each University is in the states described below.

### 2- Study population

1st to final-year medical students at the faculty of medicine were included in the study.

The study excluded members of the population who declined to consent.

### 3- Sampling

This study included first to final-year medical students from the five universities.

### 4- Sample size

The sample size was calculated for each university using the following equation:

n = N/1 + N (e) ^2.

n = Sample size.

N: Population size.

E: Level of precision.

The overall sample size of this study is 1509.

### 5- Sampling techniques

#### Cluster selection

The first stage included clustering the areas into the Khartoum state area and areas outside of Khartoum state. The second stage included a division of Khartoum state into its natural governance of Khartoum, Bahri, and Omdurman. We purposively selected Khartoum states, considering faculty size in addition to feasibility. Each university was subsequently selected from each cluster.

#### Population selection

Following calculation of the sample size in each university, students were stratified according to the population in each grade to ensure representation.

The example below demonstrates the method applied to University of Khartoum students. The example contains the total sample size, the population of each batch, the percentage of each batch within the total population of the faculty students (signified by the brackets). We calculated the sample size from each batch using this percentage.

#### **Example**

Total sample size in University of Khartoum = 399.

1st year: 333 (16.7%) = 67.

2nd year: 300 (15.2%) = 61.

3rd year: 352 (17.8%) = 71.

4th year: 314 (15.8%) = 63.

5th year: 323 (16.3%) = 65.

6th year: 361 (18.2%) = 72.

We employed a systematic random sampling method to select participants from the sampling frame of each grade. We conducted a simple random replacement from the sampling frame when a respondent declined to consent.

1176 students finally participated in the study, giving us a response rate of 77.9%.

### 6- Data Collection

We collected data using an online Google form using a self-administered questionnaire for five months. The authors developed the questionnaire according to their medical education context.

### 7- Tools and measurements

We conducted a pilot study before the primary research to ensure that questions were clear and understood appropriately. Below are the four main areas targeted by the questionnaire:

1-Demography: Demographic information included gender, university, and academic year.

2- Knowledge measurement: Categorized participants’ knowledge regarding global health as a general concept as poor or good. The knowledge was assessed by definition, general technical aspects, and general knowledge aspects within the global health arena.

3- Attitude measurement: Assessed participants’ attitudes towards global health. This was measured by assessing to what extent they believe global health is relevant and essential, receptivity to its inclusion as part of the academic curriculum, and their interest and willingness to have global health as part of their future career.

4- Practice: Reflection of student current practices and involvement in extracurricular activities related to global health.

### 8- Data analysis tools

The data collected were entered into Microsoft Excel database and analyzed using SPSS v.25 system.

Descriptive analysis was done for sociodemographic data, Knowledge questions and practice.

Attitudes were assessed using Likert scale and mean was calculated.

Associations were tested using Chi-square test; significant association when p-value < 0.05.

### 9- Ethical consideration

#### Ethical approval

has been obtained from the IRB of the Community Medicine Department- Faculty of Medicine -University of Khartoum. (No. 2/2019). Consent was also obtained from Deans of Faculties. Informed consent was obtained from participants through the google form with full explanation that they were under research study, those who refused didn’t fill the questionnaire. No information that discloses the identity of the participant was recorded by ensuring the anonymity of the research participant.

### Patient and public involvement

Patients or the public WERE NOT involved in our research’s design, conduct, reporting, or dissemination plans.

## Results

### Socio-demographics of participants

A total of 1176 medical students from five Sudanese Universities participated in the study for a response rate of 77.9%. Data was collected from November 2019 until April 2020.

Slightly more than two-thirds of all respondents were female (63.4%, N 746), and the remaining were males (36.6%, N, 430). Students from all grades within the faculty were chosen. The highest frequency was among third-year students, and the lowest was in the sixth year. The frequency varies among the universities, with the highest at Khartoum University, and the lowest at Kordofan University. (Table 1)

**Table 1:** shows the characteristics of participants (n = 1176)

Item	Description	Frequency	Percentage
Sex	Male Female	430 746	36.6% 63.4%
University	Khartoum Gezira Omdurman Islamic Alzaiem Alazhari Kordofan	337 247 262 237 93	28.7% 21% 22.3% 20.2% 7.9%
Academic year	First year Second year Third year Fourth year Fifth year Sixth Year	234 245 259 189 174 75	19.9% 20.8% 22% 16.1% 14.8% 6.4%

### Students’ knowledge regarding global health

There were nine knowledge questions to capture the awareness of Sudanese medical students regarding general concepts in the field of global health (Table 2). We utilized a simple scoring method. Each question was given one degree for the correct answer and a zero degree for the wrong answer. The poor level was determined by getting three or fewer degrees, the moderate level by getting (4–6) degrees, and the good level of knowledge by getting more than 6 degrees. By applying this method of calculation, we have quantified the level of knowledge among the respondents.

Overall, slightly less than three-fourths of respondents (72%, N, 852) were categorized as having poor knowledge, 297 (25.3%) and 27 (2.3%) were categorized as moderate and good, respectively. This gave a mean level score of 2.63 (1.63 SD), which is indicative of the generally poor knowledge among the undergraduate students.

Most students, 798 (67.9%), correctly responded to the meaning of Universal Health Coverage (UHC), and more than half of them, 653 (55.5%) correctly responded to the definition of global health. Students skewed towards the “I don’t know” option in most questions rather than giving an incorrect answer.

**Figure 1:**
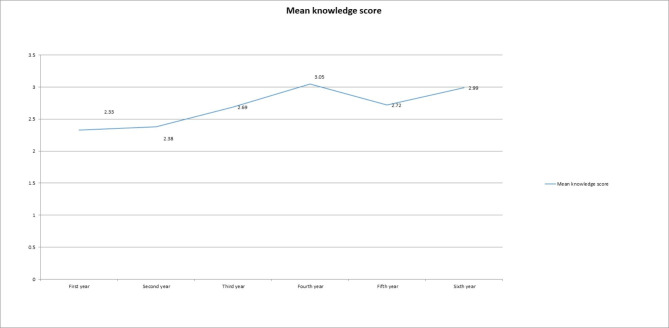
Shows the mean knowledge score of medical students in each grade, Sudan 2019–2020 (n = 1176). The mean knowledge score is highest among fourth-year medical students, while it is lowest among first-year medical students. In general, seniors’ mean knowledge score values are higher than the mean values of juniors (first, second and third years).

However, the mean knowledge score differs by grade and positively correlates with the student’s grade, where it is lowest in the first year and highest in the fourth year, when it plateaus (Fig. 1). Other significant associations were between the level of knowledge and student interest in global health (P-value 0.000). No other associations were found.

**Table 2:** shows medical students` responses to knowledge questions on global health general concepts, Sudan 2019–2020

Question	N	Correct answer	Wrong answer	Does not know
Which of the following is closest to the definition of global health?	1176	55.5%	19.4%	25.1%
What is the model that we use to analyse and understand determinants of health?	1176	24.3%	34.8%	40.9%
Which of the following is the least important to global health?	1176	13.2%	52.6%	34.3%
The “double yoke” or “double burden” of disease is most predominant in which group of countries?	1175	15%	28.1%	56.9%
Which of the following cannot be measured by DALYs (Disability-adjusted life years)?	1176	9.1%	42.6%	48.3%
What does universal health coverage (UHC) mean?	1176	67.9%	32.1%	0%
By 2015, the following has been named as the key set of global goals by the United Nations	1176	20.2%	16.3%	63.5%
Regarding whom influences global health (Major players)?	1176	37.3%	16.9%	45.7%
Which phenomenon makes global health of increasing importance nowadays?	1176	20.9%	41.9%	37.2%

### Attitudes towards global health

Results in attitude are demonstrative of the interest of medical students in global health, their perspectives on the importance and relevance of global health to medicine. It reflects what degree students agree on the importance of receiving teaching and learning opportunities in this field, and the inclusion of global health as part of their official medical education curriculum.

Table 3 summarizes the attitude level among the students. The attitude level does not correspond to knowledge results. Slightly less than half of the respondents considered global health as part of their future, 550 (46.8%). A significant association between students’ interest in global health and their agreement on including global health in the official curriculum was evident (P-value 0.00). No other associations were significant.

**Table 3:** reflects medical students’ attitudes towards global health, Sudan 2019–2020 (n = 1176)

Questions	N	Minimum	maximum	Mean	Standard deviation (SD)
Rate the importance of global health	1176	1	5	4.46	0.87
Rate the relevance of global health to medicine	1176	1	5	4.17	0.95
Is it important for students to receive learning and teaching opportunities on global health	1176	1	5	4.20	0.98
Should global health be part of the official curriculum (Medical Education curriculum)	1176	1	5	3.91	1.09
Are you interested in global health	1176	1	5	3.47	1.30

### Practice towards global health related activities

Practice questions targeted the various extracurricular activities in the undergraduate life of the medical students that relate to global health, global health conferences, courses, medical missions, research, and student exchange. Table 4 provides a summary of these activities.

Practice in global health-related extracurriculars is deficient in frequency overall. We found a significant association between considering global health as a component of a student’s future career and their participation in student exchange (p-value 0.002). No other associations were significant.

**Table 4:** reflects global health-related activities that students participated in:

Item	Frequency (%)
Have you attended any global health courses?	123 (10.5%)
Have you attended any global health conferences?	122 (10.4%)
Have you attended any activities related to global health?	459 (39%)
Have you participated in the IFMSA exchange?	92 (7.8%)
Have you conducted or participated in any global health research?	118 (10%)
Have you organized any activities related to global health (session, conference, medical mission, etc.)	321 (27.3%)

## Discussion

The prominence of this research lies in the illustration of the global health education gap in selected African medical education curriculums. Findings from this study may assist in guiding the construction of a well-structured and meaningful integration of global health in the official medical education curriculum. Schools should devote core curricular time to global health education in order to train advocates who are aware of local and global discrepancies in healthcare access and health outcomes, as well as their roles in improving the health of vulnerable populations. Health professional’s education is a system that overlaps with the health system it seeks to serve [19]. The Lancet Commission in 2010 further elaborated upon the education and health system nexus. A balance between the two systems is crucial for efficiency, effectiveness, and equity [20].

The Commission illuminated the profound influence educational institutions have in transforming health systems. After the discovery of germ theory, the beginning of the 20th century witnessed many reforms in professional education. These dramatic reforms catalyzed health gains in the past century. The 21st century is an epochal period resulting from the technological revolution, resulting in a globalized world with many implications in many spheres at the global, national, local, and individual level. The adoption of education systems should be done to the same extent as these massive changes.

Countries fail to overcome dysfunctional and inequitable health systems due to rigid curriculum, static pedagogy, insufficient adaptation to local contexts, and commercialism in the professions [20]. A brief example at the local level is the chronic gross gap between the supply and demand of the health workforce. The ratio of doctors to medical schools is 2.36 per 1000^21^, which, despite the increasing number of medical schools and medical graduates, has not been mitigated. It is noted that such problems are generally most severe in Sub-Saharan Africa [21].

To our knowledge, this is the first study of medical students’ attitudes, knowledge, and practice regarding global health in North Africa and the Middle Eastern region, and Sudan. As discussed, most of the literature is dominated by high- and upper-middle-income institutions. On average, attitude scored the highest, followed by practice and knowledge.

Overall, knowledge regarding global health is poor. The questions that scored best were only those regarding the definition aspects, namely global health and UHC, which were the only questions that more than 50% of the respondents answered correctly. The specific technical aspects (knowledge questions 2–4) were the questions that scored lowest, with the lowest score (9.1% answering correctly) witnessed in the DALY-related (Disability-Adjusted Life Year) question. Despite the excellent advocacy and the current trajectory in global health towards achieving the Sustainable Development Goals, 63% mentioned they did not know of them. In addition, most respondents answered “I don’t know” when asked about the influencers in global health. Most of them did not recognize globalization as a phenomenon requiring global health as an important discipline. This reveals the diminished general knowledge among the respondents. There was no significant disparity in scores between the different universities. However, the knowledge score seems to increase with the medical student’s grade. The mean knowledge score is highest among fourth-year medical students, 3.05 (1.69 SD), and is lowest among first-year medical students, 2.33 (1.57 SD). It is possible to logically attribute the low knowledge scores to the lack of global health exposure in the medical curricula. The study from IFMSA reports very low teaching exposure in undergraduate curricula in LMICs. Participants from LMICs were less likely to report on quality and access to healthcare based on social determinants of health. [16] It is possible that mitigating this knowledge gap could be obtained through increased curricular exposure and, at best, inclusion as a scholarly discipline in undergraduate education with elective opportunities to engage students in clinical practice and research in international settings. The scores in knowledge significantly contrasted with the attitude scores. Knowledge levels did not impact the respondents’ interest and perspectives towards global health as a discipline. This pattern is consistent with other studies in LMICs, namely in India [17] and Nigeria [18], where the low levels of knowledge did not impact students’ willingness to volunteer their experiences in international settings. This contrasts with the literature in HICs [8–11] whereby first-hand exposure seems to be a determining factor in affecting students’ interests. For example, the sharp divide in our study, in which 46.8% of participants are considering global health as a component of their future careers, with the abundance of literature in High-income countries that stated first-hand experience was associated with increased interest. This overall puts into perspective that the existing realities in LMICs could have an impact as experiences abroad expand physicians’ skills and expand their knowledge. In addition, results showed a significant association between considering global health as a future career and those exposed to international settings in HIC during IFMSA exchanges (p = 0.002). This correlates with other research findings that first-hand global health education is impactful in career choices. However, it is inconsistent with other findings that question the origin of interest in obtaining a global health career and does not attribute it to global health education. [13].

The well-known ‘Revolution of Higher Education’ in 1990, medical education has massively expanded, where the number of medical colleges has risen from 4 to 1990 to 34 in 2012, resulting in a sharp increase of medical graduates, from 2499 to 1989 to 12,140 in 2008. Currently, strengthening public health education is identified as a priority by Sudan’s Minister of Health and Higher Education. Attrition of medical practitioners yet remains a challenge. However, it can be argued that outside experience provides flexibility of thinking, valuable ‘outsider’s’ perspective that is equivalent to global health electives conducted in developing countries. As a result, global health education may provide great value compared to developed countries electives, Destinations of Sudanese physicians’ migration are respectively in Saudi Arabia, Gulf States, the United Kingdom, the Republic of Ireland and the United States [22]. Moreover, our results showed a strong association between interest in obtaining a global health career, level of knowledge, and its inclusion as part of the official curriculum. Students recognize the gaps in their medical education and will be receptive to changes in the academic environment and exposure to global health education. Robust findings were that most of the participants thought global health is very relevant to medicine, that it is vital to receive learning and teaching opportunities in global health, and that they strongly agreed for it to be included in the official curriculum. This is consistent with many studies recommending the inclusion of global health in medical education. There is clearly a gap in global health education in medical schools, and current teaching does not correspond to what medical students want [15].

As discussed above, there is an association between participating in IFMSA exchanges and global health career interest. However, there is no significant association between considering global health as a career and conducting or participating in global health research (p = 0.2). This is contrary to findings in Cox’s cross-sectional study [13]. No other associations were significant regarding the practices.

The findings of our study must be seen in the light of some limitations. The concept of the research, although important, is relatively new; hence, no previous questionnaire was validated, and as a result, the reliability of the questionnaire couldn’t be checked. The findings from this study may apply to other medical schools outside of Sudan, but transferability may not be possible outside of northern African medical education. The findings from our research used an audit trail. All raw data, coding strategies, databases, and statistical analysis were verified and checked for interpretation by the researcher’s supervisors. We believe other researchers could confirm the results of our study based on our data. We recommend starting with a qualitative study to reflect on the general concept of global health that students perceive.

## Conclusion

As global health increases in importance, global health education in undergraduate medical education will continue to be a subject of discussion. A low level of knowledge was demonstrated among the participants in this study, which has been attributed to the lack of global health exposure in the medical curriculum. On the other hand, community-based education helped graduates to work in their local communities.

Without inclusion efforts, another generation of doctors will be graduate with a narrow, national outlook. Global health knowledge will equip graduates to handle the complex challenges of the 21st century (such as antimicrobial resistance, health workforce migration, access to medicines, etc.) encountered globally, in their national health systems, and in their own professional lives. In addition, it has the added benefit of increasing career interest among students.

This study found that the younger generation’s attitudes are interconnected, and they are aware of the changes required in their education that will qualify them to become doctors in the 21st century.

## Data Availability

All data relevant to the study are included in the article. **Authors contributions**. Sawazen Malik and Asi Alkoronky were involved in the conception of the research, study design, acquisition of data, and drafted the manuscript. Mojahid, Safaa, Fares and Rawasi have substantially contributed to the acquisition of data and manuscript writing. Daffalla Alam Elhuda has contributed to the research design and in critical revision of the work. All authors have approved the final version to be published. All authors agree to be accountable for all aspects of the work in ensuring that questions related to the accuracy or integrity of any part of the work are appropriately investigated and resolved.
